# FlexGraph: Flexible partitioning and storage for scalable graph mining

**DOI:** 10.1371/journal.pone.0227032

**Published:** 2020-01-24

**Authors:** Chiwan Park, Ha-Myung Park, U. Kang

**Affiliations:** Department of Computer Science and Engineering, Seoul National University, Seoul, Republic of Korea; Singapore University of Technology and Design, SINGAPORE

## Abstract

How can we analyze large graphs such as the Web, and social networks with hundreds of billions of vertices and edges? Although many graph mining systems have been proposed to perform various graph mining algorithms on such large graphs, they have difficulties in processing Web-scale graphs due to massive communication and I/O costs caused by communication between workers, and reading subgraphs repeatedly. In this paper, we propose FlexGraph, a scalable distributed graph mining method reducing the costs by exploiting properties of real-world graphs. FlexGraph significantly decreases the communication cost, which is the main bottleneck of distributed systems, by exploiting different edge placement policies based on types of vertices. Furthermore, we propose a flexible storage format to reduce I/O costs when reading input graph repeatedly. Experiments show that FlexGraph succeeds in processing up to 64× larger graphs than existing distributed memory-based graph mining methods, and consistently outperforms previous disk-based graph mining methods.

## Introduction

How can we analyze enormous networks like the Web and social networks which have hundreds of billions of vertices and edges? Graph mining algorithms such as shortest path computation, PageRank, connected component computation, and random walk with restart enable many network analyses. Much effort has been devoted to developing scalable distributed graph processing systems that provide simple primitives to represent such graph mining algorithms [1–5]. Due to the simplicity of the primitives, the systems have been used in numerous applications such as radius estimation [6, 7], spectral analysis [8, 9], pattern recognition [10], recommender systems [11], community detection [12, 13], visualization [14, 15], and clustering [16].

Most of the distributed graph mining systems, however, have problems in handling a very large graph because of massive communication and I/O costs. The massive communication cost is the main factor impeding the scalability of distributed graph mining systems, which are grouped into memory-based and disk-based ones. Memory-based systems including Pregel [4], PowerGraph [5], and GraphX [17] load input graph into distributed memory, and communicate through the memory directly. If the graph and the intermediate data for communication do not fit into the distributed memory, the systems will fail. Disk-based systems such as Hama [18], and PEGASUS [1] increase their scalability by exploiting distributed file systems like HDFS [19] along with local file system of each machine. However, these systems also cannot handle very large graphs as they require a lot of communication through network and disk I/Os, which are well-known causes of performance degradation. There have also been attempts to reduce the communication cost to increase the scalability like UNICORN [20], HybridGraph [21], and Pregelix [22], but they focus only on the communication cost; they have other performance bottlenecks like inefficient data structures for loading graph, and fail to process enormous graphs. Thus, it is desired to shrink the I/O cost in designing a distributed graph mining system.

In this paper, we propose FlexGraph, a new scalable graph processing method on distributed systems, utilizing real-world graph properties to reduce communication and I/O costs dramatically. FlexGraph has two main ideas, flexible edge placement and storage format, to reduce the communication cost and the I/O cost caused by inefficient data format. We observe that a large portion of the communication cost is caused by high degree vertices. Our method processes data from high degree vertices on the same machine so that a large amount of data can be aggregated before communication. Furthermore, our method exploits a flexible storage format which is an efficient storage format for partitioned subgraphs, to reduce I/O cost caused by loading graphs in iterative computation. Thanks to the significantly reduced costs, FlexGraph succeeds in processing billion-scale graphs, which all other state-of-the-art distributed systems fail to process. Our main contributions are as follows:

- **Method**. We propose FlexGraph, a new scalable distributed graph mining system, which dramatically reduces the communication cost by specially handling high-degree vertices. We apply an efficient storage format to further reduce the I/O cost caused by loading subgraphs repeatedly.
- **Cost analysis**. We give theoretical analyses of the proposed method in terms of the communication cost.
- **Performance**. We empirically evaluate FlexGraph using both large real-world and synthetic networks. We emphasize that only our system succeeds in processing ClueWeb12, the largest public graph, which has 6 billion vertices and 71 billion edges.

The rest of the paper is organized as follows. We review existing large-scale graph processing systems, discuss communication and I/O costs of distributed graph processing systems, and introduce the GIM-V primitive. We then describe the proposed method FlexGraph in detail. After showing experimental results, we conclude this paper. The symbols frequently used in this paper are summarized in Table 1. The codes and datasets used in this paper are publicly available at https://github.com/snudatalab/FlexGraph.

**Table 1:** Table of symbols.

Symbol	Description
*v*	Vector, or set of vertices
*θ*	Degree threshold to divide low and high out-degree vertices
*out*(*p*)	Set of out-neighbors of a vertex *p*
*b*	Number of vector blocks or vertex partitions
*ψ*	Vertex partitioning function: *p* → {1, …, *b*}
*v*_*i*_	*i*-th element of *v*
*v*^(*i*)^	Set of vector elements (*p*, *v*_*p*_) where *ψ*(*p*) = *i*
$v_{s}^{\left(i\right)}$	Set of vector elements (*p*, *v*_*p*_) ∈ *v*^(*i*)^ where |*out*(*p*)| < *θ*
$v_{d}^{\left(i\right)}$	Set of vector elements (*p*, *v*_*p*_) ∈ *v*^(*i*)^ where |*out*(*p*)| ≥ *θ*
|*v*|	Number of non-zero elements in *v*
*M*	Matrix, or set of edges; each column and row represent a source and a destination vertex, respectively.
*m*_*i*,*j*_	(*i*, *j*)-th element of *M*
*M*^(*i*,*j*)^	Set of matrix elements (*p*, *q*, *m*_*p*,*q*_) where *ψ*(*p*) = *i* and *ψ*(*q*) = *j*
$M_{s}^{\left(i,j\right)}$	Set of matrix elements (*p*, *q*, *m*_*p*,*q*_) ∈ *M*^(*i*,*j*)^ where |*out*(*q*)| < *θ*
$M_{d}^{\left(i,j\right)}$	Set of matrix elements (*p*, *q*, *m*_*p*,*q*_) ∈ *M*^(*i*,*j*)^ where |*out*(*q*)| ≥ *θ*
|*M*|	Number of non-zero elements in *M* (= number of edges in a graph)
⊗	User-defined matrix-vector multiplication

## Background and related work

In this section, we first review representative graph processing systems, and show their limitations on scalability. Then, we discuss communication and I/O costs of the distributed graph mining systems according to graph partitioning schemes and storage formats. Finally, we introduce the details of the GIM-V primitive focusing on the matrix-vector representation for graph algorithms.

### Large-scale graph processing systems

Large-scale graph processing systems perform various graph algorithms on directed graphs containing many vertices and edges. The systems can be classified into three groups: single-machine based systems, memory-based distributed systems, and disk-based distributed systems.

Single-machine graph mining systems [23–26] handle large graphs with external-memory (i.e., disk) and optimize disk I/O cost to achieve high performance. Some single-machine systems [27–29] use specialized hardwares like NVMe SSDs or GPUs to improve performance. However, all of these systems have limited scalability as they use only a single machine.

A typical approach to handle large-scale graphs is using memory on distributed workers. Recently, several memory-based distributed graph processing systems have been proposed: Pregel [4], PowerGraph [5], GraphX [17], Presto [30], PowerLyra [31], GraphFrames [32], and GRAPE [33, 34]. Even though these distributed-memory systems achieve faster performance than single-machine systems do, they cannot process graphs larger than the size of the distributed memory. As reported by Sahu et al [35], facing performance degradation or out-of-memory error is a common challenge of memory-based distributed systems.

To overcome this limitation, some graph processing systems provide out-of-core support using disks of distributed workers. Pregelix [22] succeeds in processing graphs whose sizes exceed the distributed-memory size by exploiting multiple join operations and out-of-core support of Hyracks [36], a general data processing engine. However, in our experiments, Pregelix fails to process graphs with highly skewed degree distribution due to space overhead of join index structures. HybridGraph [21] stores the input graph and the intermediate data on disks for better scalability. However, the performance of these systems is degraded because of two main reasons: they suffer from massive disk I/Os caused by an inefficient storage format for the partitioned subgraphs in local file system, and they do not fully exploit memory space in each worker. MapReduce-based systems have been also proposed as MapReduce [37] is a scalable disk-based distributed processing framework. PEGASUS [1] and GBASE [38, 39] are representative MapReduce-based graph mining libraries based on a generalized matrix-vector multiplication. SGC [40] is another MapReduce-based system exploiting two join operations, namely NE join and EN join. The MapReduce-based systems, however, still have limited scalability because they need to shuffle the input graph repeatedly. UNICORN [20] avoids massive data shuffling by exploiting HBase, a distributed database system on Hadoop, but it reaches another performance bottleneck, intensive random accesses to HBase.

### Costs of distributed graph processing systems

Many distributed graph processing systems we discussed in the previous section have similar processing steps. The systems first partition the graph into multiple subgraphs to spread the work across multiple workers. Then, a graph program implemented on message passing primitives is executed in parallel. The message passing primitives are used to represent graph algorithms as iterative message passing among vertices; each vertex has its own value which is updated by aggregating the values from the incoming neighbors at each iteration. The major performance bottlenecks of distributed graph processing systems are: (1) communication cost between workers, and (2) I/O cost to read the partitioned subgraphs.

#### Communication cost between workers

**Fig 1:**
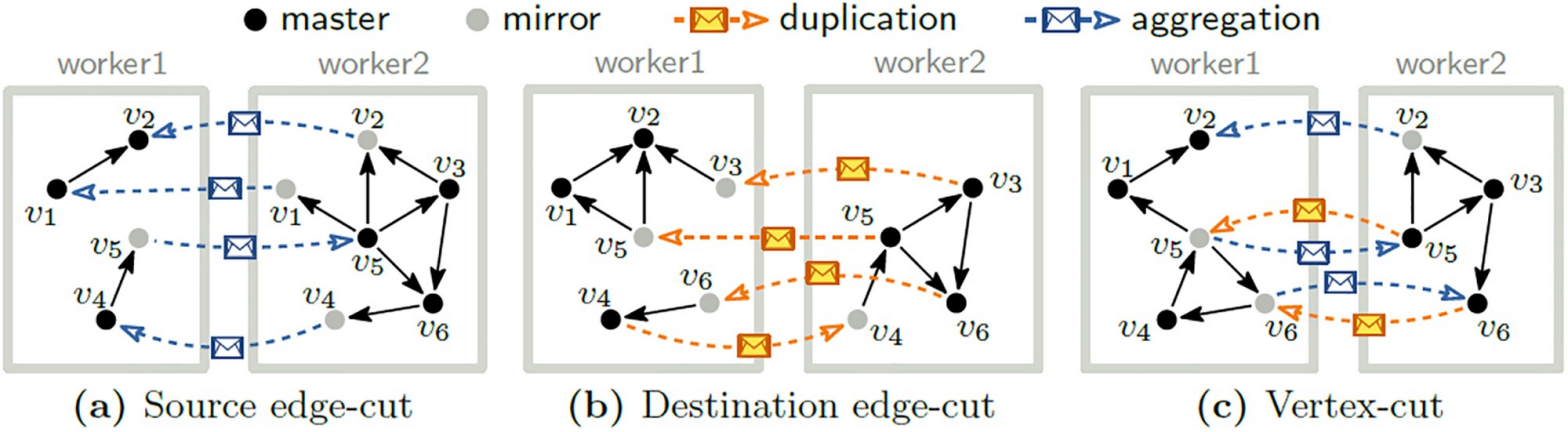
Examples of communication patterns according to graph partitioning methods: Edge-cut and vertex-cut. Workers exchange two types of messages: one for duplicating the value of a vertex to its mirrors and the other for copying the partially aggregated value of a mirror to its master. The sum of all exchanged messages is the communication cost. Note that the edge-cut methods also create the mirrors to aggregate duplication/aggregation messages from/to the same vertex even the edge-cut methods cut edges instead of vertices.

**Fig 2:**
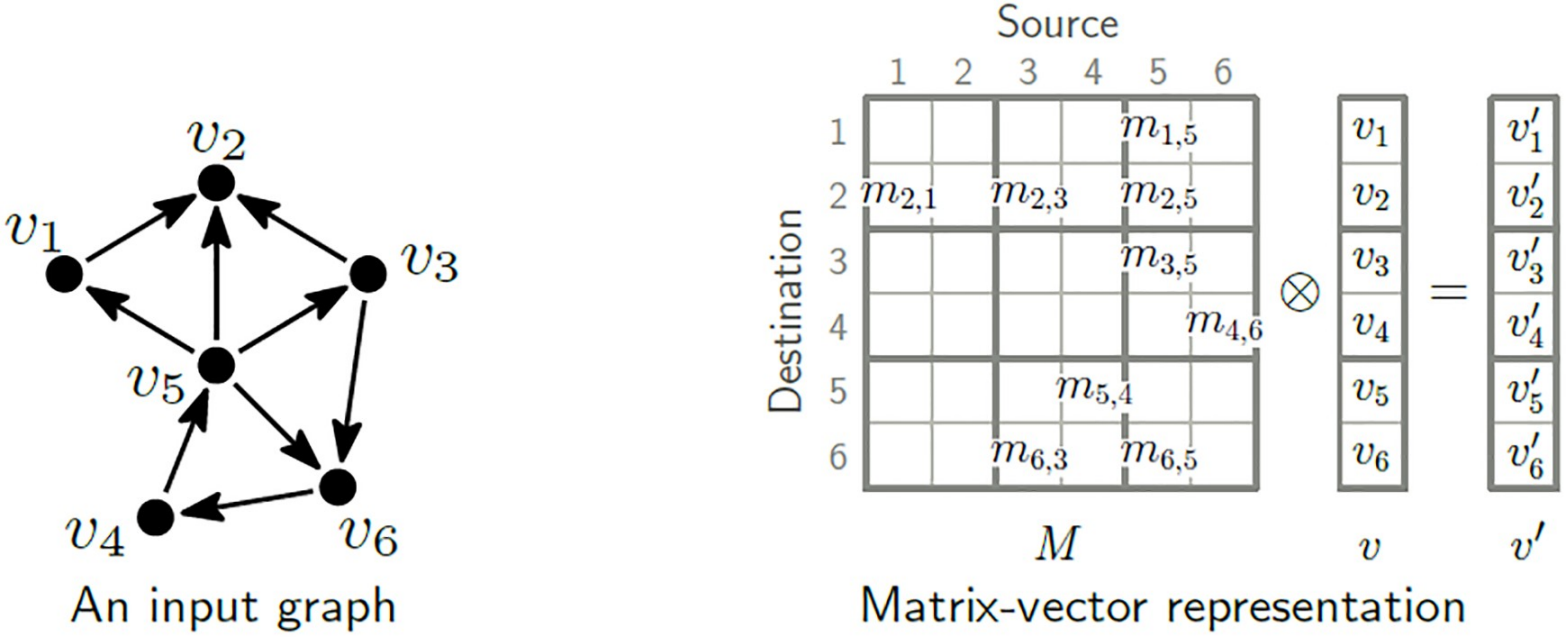
Matrix-vector representation of a directed graph. The matrix *M* and the vector *v* correspond to the edge set and the vertex set of the graph, respectively. An iteration of graph processing is represented as a matrix-vector multiplication with generalized operations.

The communication cost between workers depends on the graph partitioning methods. We introduce two representative graph partitioning methods: vertex-cut and edge-cut, and analyze communication patterns and costs of them. Fig 1 shows examples of communication patterns using edge-cut and vertex-cut given the graph in Fig 2. Both methods assign each vertex to a worker using vertex partitioning function. In general, random hash function is used for the vertex partitioning function to achieve small graph loading time. In the example, both edge-cut and vertex-cut assign vertices *v*_1_, *v*_2_, and *v*_4_ to worker 1 and assign vertices *v*_3_, *v*_5_, and *v*_6_ to worker 2. Edge-cut and vertex-cut assign edges differently. Edge-cut splits a graph into subgraphs along edges by assigning an edge to the worker where the source vertex (or the destination vertex) of the edge is assigned. Thus, there are two types of edge-cut: source edge-cut and destination edge-cut. Fig 1a and 1b show examples of edge-cut when edges get together on source vertices (source edge-cut) or on destination vertices (destination edge-cut). On the other hand, vertex-cut splits a graph into subgraphs along vertices by assigning an edge to any worker regardless of where the two end vertices of the edge are assigned. In Fig 1c, for example, edge (*v*_5_, *v*_6_) is assigned to worker 1 although its connected vertices *v*_5_ and *v*_6_ are in worker 2.

Regardless of the partitioning methods, if an edge is assigned to a worker but a vertex incident to the edge is assigned to a different worker, we make a replica (i.e., mirror) of the vertex in the worker. For example in Fig 1a, we make a replica of *v*_5_ in worker 1 since edge (*v*_4_, *v*_5_) is in worker 1 but vertex *v*_5_ is in worker 2. We call a vertex that is not a mirror a master.

Graph processing systems have different communication patterns depending on the graph partitioning method used. The next value of a vertex is computed by aggregating the messages from the incoming neighbors of the vertex. A system using source edge-cut needs network communication to compute the next value of a vertex because mirrors of the vertex in other workers have to send the aggregated messages to the master of the vertex. In Fig 1a, the next value of *v*_2_ is computed from *v*_2_’s incoming neighbors *v*_1_, *v*_3_, and *v*_5_, but the aggregated messages from *v*_3_ and *v*_5_ are in worker 2; thus, worker 1 receives the aggregated messages of *v*_3_ and *v*_5_ from worker 2 as *aggregation* messages. Similarly, a system using destination edge-cut also requires network communication to compute the next value of a vertex because the value of incoming neighbors should be copied from other workers. In Fig 1b, the next value of *v*_4_ is computed from *v*_4_’s incoming neighbor *v*_6_ which is in worker 2; thus, worker 2 sends the value of *v*_6_ to worker 1 as *duplication* messages.

Systems using vertex-cut use both types of messages, while systems using edge-cut use only one type of messages. In Fig 1c, the next value of *v*_2_ is computed by transferring an aggregation message from the mirror of *v*_2_. On the contrary, the next value of *v*_4_ is computed by transferring a duplication message of *v*_6_. The communication cost between workers is the sum of all exchanged messages. The vertex partitioning function to distribute vertices into workers also affects the communication cost because concentration of high-degree vertices incurs load balancing and network congestion issues. PowerLyra [31] proposes a new graph partitioning scheme called hybrid-cut which applies edge-cut for low in-degree vertices, and vertex-cut for high in-degree vertices, respectively, under similar intuition of our proposed method. Hybrid-cut achieves lower number of mirrors than those of the other partitioning schemes. PowerLyra focuses on replication factor which is the number of mirror vertices per master vertex. However the replication factor is not the same as the number of messages transferred between workers. For example, in PowerLyra, a high in-degree vertex produces up to 4 times more messages than a low in-degree vertex does. FlexGraph is designed to minimize the total number of messages between workers. Also, PowerLyra does not fully analyze the performance tradeoff between edge-cut and vertex-cut in its design, and does not apply techniques for disk-based systems such as block-wise operations, unlike our proposed FlexGraph.

#### I/O cost to read subgraphs

If a graph processing system uses an external storage, there is another I/O cost to read the partitioned graphs from the external storage. For example, systems like HybridGraph, GraphD [41] and Pregelix store the partitioned subgraphs in local disks of workers, and load the subgraphs in every iteration to avoid scalability issues such as out-of-memory error. Reading the subgraphs from external storage causes massive disk I/Os. We emphasize that the amount of disk I/Os caused by loading the subgraphs exceeds the communication cost between workers because the number of messages is less than the number of edges in many graph mining algorithms. Therefore, efforts to reduce the size of partitioned subgraphs is also valuable. Recently, researchers have proposed several data formats to store graphs in distributed graph systems. GBASE [38] divides an adjacency matrix into multiple sub-matrices, and applies typical compression algorithms like *Gzip* and *Gap Elias’*-*γ* encoding to the sub-matrices. However, the block compression in GBASE requires additional expensive preprocessing step such as SlashBurn [42, 43] to find homogeneous regions in the adjacency matrix. Elgohary et al. [44] proposed CLA, a compressed matrix data format that groups columns into column groups, and applies co-coding representation to each column group. However, CLA relies on high correlation between columns which is not reasonable for graph data. Liakos et al. [45, 46] proposed four compression techniques for storing out-edges of a vertex into bit-vectors to exploit locality of references in real-world graphs. However, the approaches using bit-vectors require a strong assumption that there are many out-edges connected to consecutive vertices from a vertex. This assumption is easily broken if the input graph is partitioned without source edge-cut because the out-edges of a vertex can be placed in several subgraphs. Furthermore, the source edge-cut causes massive communication cost; thus, the effect of compression using bit-vectors is decreased.

### GIM-V: Graph processing as matrix computation

GIM-V, a well-known graph mining primitive introduced in PEGASUS [1], represents graph algorithms in the form of matrix-vector multiplication. GIM-V and its variations are widely used in many graph mining systems [20, 38, 47, 48]. Fig 2 shows a matrix-vector representation of a directed graph. The matrix and the vector represent the edge set and the vertex set, respectively. The value on *i*-th column and *j*-th row of the matrix corresponds to the weight of edge (*i*, *j*) in the graph, and *i*-th value of the vector corresponds to the value of *i*-th vertex. GIM-V requires a user to describe only three operations for a graph algorithm: combine2, combineAll, and assign. Consider a matrix *M* of size *n* × *n*, and a vector *v* of size *n*, where *m*_*i*,*j*_ is the (*i*, *j*)-th element of *M*, and *v*_*i*_ is the *i*-th element of *v* for *i*, *j* ∈ {1, ⋯, *n*}. Then, the operations play the following roles:

- combine2(*m*_*i*,*j*_, *v*_*j*_): return the combined value *x*_*i*,*j*_ from a matrix element *m*_*i*,*j*_ and a vector element *v*_*j*_.
- combineAll({*x*_*i*,1_, ⋯, *x*_*i*,*n*_}): reduce the input values to a single value *r*_*i*_.
- assign(*v*_*i*_, *r*_*i*_): compute the new *i*-th vector element $v_{i}^{\prime }$ for the next iteration from the current *i*-th vector element *v*_*i*_ and the reduced value *r*_*i*_.

Let *M* ⊗ *v* be a user-defined generalized matrix-vector multiplication between the matrix *M* and the vector *v*. The new *i*-th vector element $v_{i}^{\prime }$ of the result vector *v*′ of *M* ⊗ *v* is then:

$$v_{i}^{\prime }=\text{assign}(v_{i},\text{combineAll}\left(\left\{x_{i,j}|x_{i,j}=\text{combine}2\left(m_{i,j},v_{j}\right),j\in \left\{1,\cdots ,n\right\}\right\}\right))$$

GIM-V can be identified as a message passing based graph program primitive like Gather-Apply-Scatter [5], and Signal-Collect [49]. These primitives have a similar structure: (1) multiple functions to describe a graph program, and (2) an ordering to execute the functions. We focus on GIM-V to describe our proposed method; however, the method is general enough to run on other compatible primitives. Although there are incompatible primitives such as vertex-centric [4] and partition-centric [50, 51] ones, most graph algorithms can be implemented both on GIM-V compatible and incompatible primitives. More details about graph program primitives are discussed in [52] and [53].

## Proposed method

In this section, we propose FlexGraph, a fast and scalable distributed graph mining method by reducing the communication and I/O costs in distributed graph computation. As we discussed before, reducing the costs in a distributed graph mining method is critical for improving scalability as well as speed of the method. We have the following main ideas to reduce the costs.

1. *Flexible edge placement* significantly shrinks the communication cost for transferring intermediate data by dividing vertices into high-degree vertices and low-degree vertices, and applying different edge placement methods to them.
2. *Flexible storage format* enables FlexGraph to reduce the I/O cost to read the partitioned subgraphs repeatedly.

We first introduce the key idea of the flexible edge placement with analysis of communication cost of graph computation. Then, we describe the storage format to reduce I/O cost for reading the input graph. Lastly, we discuss implementation issues of FlexGraph.

### Flexible edge placement

How can we efficiently reduce the communication cost among workers in graph processing? We note that high out-degree vertices send massive aggregation messages which cause performance degradation. As discussed in the previous section, the massive aggregation messages can be replaced with a few duplication messages by changing graph partitioning methods. Our proposed method, flexible edge placement, shrinks the number of aggregation messages by splitting the vertex set into a high out-degree vertex set and a low out-degree vertex set, and applying destination edge-cut and source edge-cut on the edges from the sets, respectively.

**Fig 3:**
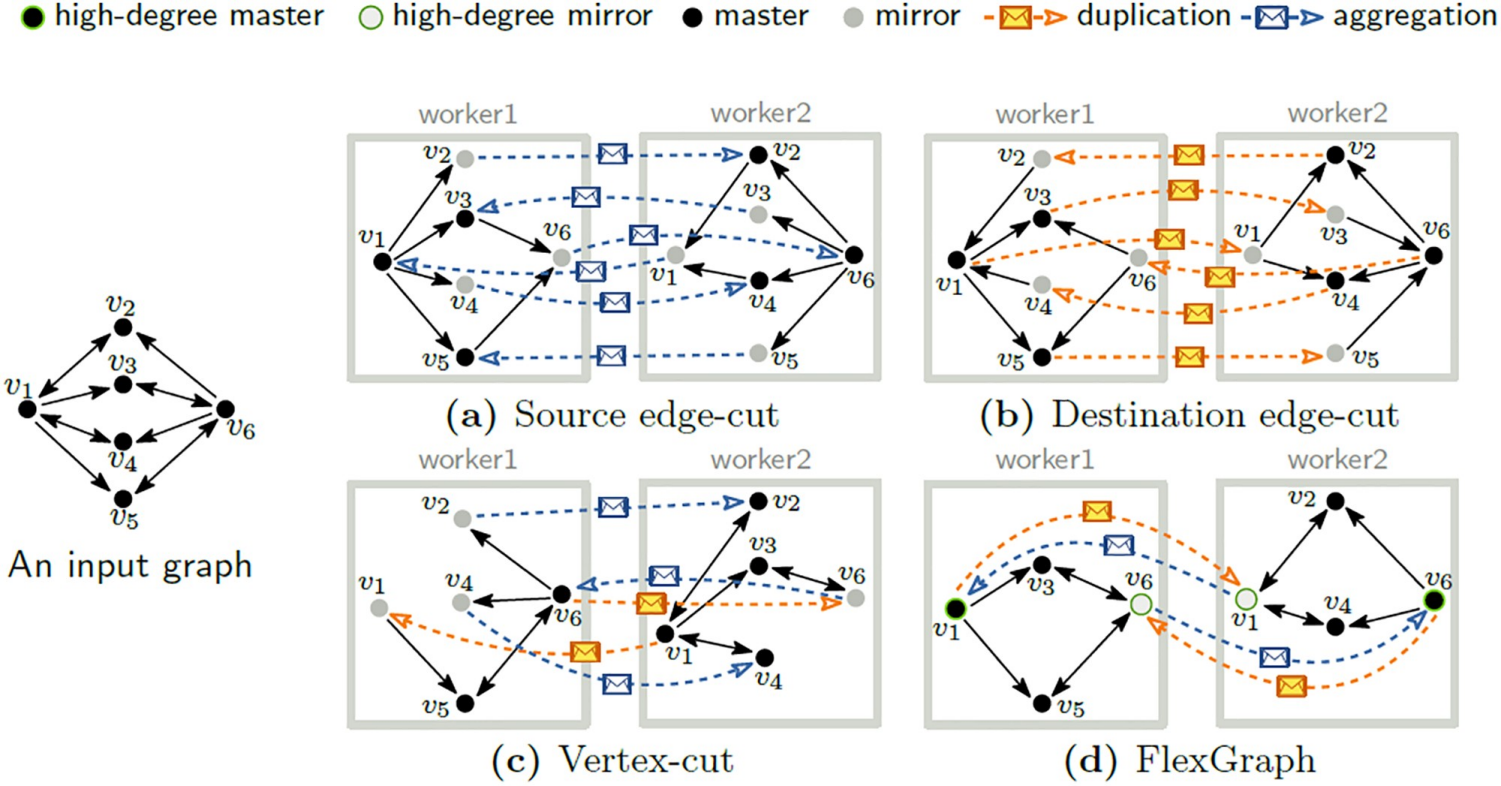
Effects of flexible edge placement. (a) If we use source edge-cut, a worker who has high out-degree vertex produces massive aggregation messages because many mirrors of outgoing neighbors are in other workers. (b) Even if we use destination edge-cut, the number of messages does not decrease much because there are many duplication messages from many mirrors of incoming neighbors in other workers. (c) Vertex-cut reduces the number of messages by reducing the number of mirrors. However, computing the optimal vertex-cut is NP-hard [54, 55], and computing an approximate solution requires shared data between workers, or massive pre-computation time [56]. (d) Our method exploits source edge-cut for low out-degree vertices to reduce duplication messages from a large number of low out-degree vertices, and destination edge-cut for high out-degree vertices to suppress aggregation messages caused by high out-degree vertices.

Fig 3 illustrates the effect of the flexible edge placement. In Fig 3a, high out-degree vertices with source edge-cut cause massive aggregation messages because the worker who has a high out-degree vertex (such as *v*_1_) creates a lot of mirrors (such as *v*_2_ and *v*_4_). Even if we use destination edge-cut as in Fig 3b, the number of messages does not decrease much because massive duplication messages are required by a whole bunch of low out-degree vertices like *v*_2_, *v*_3_, *v*_4_, and *v*_5_. Vertex-cut, as shown in Fig 3c, can reduce the number of messages by reducing the number of mirrors with both types of messages. However, computing an optimal vertex-cut for a given graph is extremely expensive. Our proposed FlexGraph takes the advantages of both types of edge-cuts by using source edge-cut on low out-degree vertices to reduce duplication messages of massive low out-degree vertices and destination edge-cut on high out-degree vertices to suppress aggregation messages from high out-degree vertices, respectively. By suppressing the effects of high out-degree vertices, FlexGraph resolves load balancing and network congestion problem incurred by concentration of high out-degree vertices due to poor vertex partition function. For example in Fig 3d, edges from high out-degree vertices whose out-degree is larger than 3 (such as *v*_1_ and *v*_6_) are distributed by destination edge-cut, while edges from low out-degree vertices like *v*_2⋯5_ are located with the source vertices by source edge-cut. As a result, the total number of messages decreases.

We verify our intuition by analyzing the communication cost depending on edge-cuts. To analyze dominant effects of communication patterns in graph processing, we consider the worst-case scenario where all vertices are always active and randomly distributed among workers. We also show that flexible edge placement works well in other scenarios in experiments section. As shown in Figs 1a and 3a, source edge-cut uses only aggregation messages for all vertices in graph. Lemma 1 states the communication cost of graph processing using only aggregation messages.

**Lemma 1**. *Graph processing using only aggregation messages has the following communication cost C*_*a*_ *per iteration*:

$$\begin{array}{c} C_{a}=\left(b-1\right)n(1-e^{-E[d_{q}]/b}) \end{array}$$ (1)

*where n is the number of vertices*, $E\left[d_{q}\right]$ *is the average out-degree of the input graph, and b is the number of vertex partitions*.

*Proof*. The communication cost of graph processing using only aggregation messages is the cost to transfer the intermediate results between workers via network. To transfer one of the intermediate results, the cost |*v*^(*i*,*j*)^| is required where *v*^(*i*,*j*)^ is the results of graph computation on subgraph *M*^(*i*,*j*)^ and *v*^(*j*)^. Therefore,

$$\begin{array}{c} C_{a}=\sum_{i\neq j}E[|v^{\left(i,j\right)}|]=b\left(b-1\right)E[|v^{\left(i,j\right)}|] \end{array}$$ (2)

where *b* is the number of vertex partitions. For each vertex *p* ∈ *v*^(*i*)^, let *X*_*p*_ denote an event that *p*’s computed value in *v*^(*i*,*j*)^ is non-zero. Assuming the vertex partition function *ψ* evenly distributes vertices into the vertex partitions, the probability of the event *X*_*p*_ is given by

$$P(X_{p})=1-P\left(p\;\text{has}\;\text{no}\;\text{in-edges}\;\text{in}\;M^{\left(i,j\right)}\right)=1-\prod_{q\in v^{\left(j\right)}}(1-\frac{d_{q}}{n})$$

where *d*_*q*_ is the out-degree of vertex *q*, and *n* is the number of vertices in graph. Considering *d*_*q*_ as a random variable whose value is from an out-degree distribution, we obtain

$$P(X_{p})=1-\prod_{q\in v^{\left(j\right)}}(1-\frac{d_{q}}{n})\approx 1-(1-\frac{E[d_{q}]}{n}{)}^{n/b}$$ (3)

Since we target billion-scale graphs, we approximate the probability by the following equation:

$$P(X_{p})=1-(1-\frac{E[d_{q}]}{n}{)}^{n/b}\approx 1-\lim_{n\to \infty }(1-\frac{E[d_{q}]}{n}{)}^{n/b}=1-e^{-E[d_{q}]/b}$$

The expected size of the intermediate results is the sum of non-zero probabilities of vertices in the results. Therefore,

$$E[|v^{\left(i,j\right)}|]=\sum_{p\in v^{\left(i\right)}}P\left(X_{p}\right)=\sum_{p\in v^{\left(i\right)}}(1-e^{-E[d_{q}]/b})=\frac{n}{b}(1-e^{-E[d_{q}]/b})$$ (4)

Combining Eqs (2) and (4), we obtain the claimed communication cost.

On the other hand, destination edge-cut uses only duplication messages for all vertices. Lemma 2 states the communication cost of graph processing using only duplication messages.

**Lemma 2**. *Graph processing using only duplication messages has the communication cost C*_*d*_ per iteration:

$$\begin{array}{c} C_{d}=\left(b-1\right)n \end{array}$$ (5)

*where n is the number of vertices and b is the number of vertex partitions*.

*Proof*. If we use only duplication messages, each worker should load all vertex partitions except one from other workers. This causes (*b* − 1)*n* communication cost. Note that the graph processing using only duplication messages assumes that an edge is assigned to the worker that has the destination vertex of the edge, and the intermediate result can be consumed in the same worker which creates the result.

Lemmas 1 and 2 state that the communication cost depends on the number of vertices, the average out-degree of the input graph, and the number of vertex partitions. The lemmas indicate that if the input graph has a small number of vertices and the average out-degree is huge, graph processing using only duplication messages requires less communication cost than that using aggregation messages. In the opposite case, using only aggregation messages results in less communication cost than using only duplication messages.

From this observations, the flexible edge placement divides the vertex set *v* into multiple partitions *v*^(*i*)^ using a user-defined partitioning function *ψ* (i.e., *ψ* = a random hash function). In other words, the master vertex of each vertex is assigned to a worker by the partitioning function. Then, for each vertex partition *v*^(*i*)^, the method further divides the partition into two subsets: sparse vertex set $v_{s}^{\left(i\right)}$ and dense vertex set $v_{d}^{\left(i\right)}$. The out-degree of all vertices in the sparse vertex set is less than a given threshold *θ*, while the out-degree of all vertices in the dense vertex set is greater than or equal to *θ*. After that, the edge set *M* is partitioned into multiple partitions *M*^(*i*,*j*)^ which contains the edges from the vertices in *v*^(*j*)^ to the vertices *v*^(*i*)^. Like the vertex partitions, each edge partition *M*^(*i*,*j*)^ is divided into two subsets: (1) the sparse edge set $M_{s}^{\left(i,j\right)}$ that contains the edges from vertices in the sparse vertex set $v_{s}^{\left(j\right)}$, and (2) the dense edge set $M_{d}^{\left(i,j\right)}$ that contains the edges from vertices in the dense vertex set $v_{d}^{\left(j\right)}$. The edge sets are grouped and assigned into a worker depending on the type of partitions. The sparse edge set $M_{s}^{\left(:,j\right)}$ is grouped with other sparse edge sets which share the same source vertex set *v*^(*j*)^, and assigned to the worker which has *v*^(*j*)^. The dense edge set $M_{d}^{\left(i,:\right)}$ sharing the same destination vertex set *v*^(*i*)^ is grouped with, and assigned to the worker who has *v*^(*i*)^. Therefore, only aggregation messages are incurred to process the sparse edge set $M_{s}^{\left(:,j\right)}$, while only duplication messages are incurred to process the dense edge set $M_{d}^{\left(i,:\right)}$.

Then, how can we choose the out-degree threshold *θ* to classify the high out-degree vertices and the low out-degree vertices? The communication cost significantly depends on *θ*. If we set *θ* = 0, the method uses only duplication messages because there is no vertex in the sparse vertex sets. On the other hand, if we set *θ* = ∞, the method uses only aggregation messages because there is no vertex in the dense vertex sets. To find the best *θ*, we present the communication cost *C*_*flex*_ of FlexGraph in Lemma 3; FlexGraph chooses *θ* that minimizes *C*_*flex*_.

**Lemma 3**. *Graph processing using the flexible edge placement has communication cost C*_*flex*_ *per iteration*:

$$\begin{array}{c} C_{flex}=\left(b-1\right)n(1-e^{-E[d_{q}\left(\theta \right)]\cdot (1-P_{out}\left(\theta \right))/b}+P_{out}\left(\theta \right)) \end{array}$$ (6)

*where n is the number of vertices in the input graph, d*_*q*_(*θ*) *is the average out-degree of vertices whose out-degree is less than θ, b is the number of vertex partitions, and P*_*out*_(*θ*) *is the ratio of vertices whose out-degree is greater than or equal to θ*.

*Proof*. The communication cost *C*_*flex*_ of graph processing is the sum of the number *C*_*a*_ of aggregation messages caused by the vertices in the sparse vertex sets, and the number *C*_*d*_ of duplication messages caused by the vertices in the dense vertex sets. From Eq (3), we get:

$$\begin{array}{c} P\left(X_{p}\right)=1-{\left(1-\frac{E[d_{q}\left(\theta \right)]}{n}\right)}^{n\cdot (1-P_{out}\left(\theta \right))/b} \end{array}$$

by substituting the average out-degree *d*_*q*_(*θ*) in sparse vertex set for *d*_*q*_ and the number *n* ⋅ (1 − *P*_*out*_(*θ*))/*b* of vertices in sparse vertex set for |*v*^(*j*)^| which is *n*/*b* in Eq (3). By applying similar steps as in Lemma 1, we get:

$$\begin{array}{c} C_{a}=\left(b-1\right)n(1-e^{-E[d_{q}\left(\theta \right)]\cdot (1-P_{out}\left(\theta \right))/b}) \end{array}$$

as the communication cost caused by sparse vertex sets and sparse edge sets. To compute the number *C*_*d*_ of duplication messages in the flexible edge placement, we need to compute the number *n*_*d*_ of vertices in dense vertex sets because only high out-degree vertices whose out-degree is greater than or equal to *θ* produce duplication messages. Therefore, we get:

$$\begin{array}{c} n_{d}=n\cdot P_{out}\left(\theta \right) \end{array}$$ (7)

where *P*_*out*_(*θ*) is the ratio of vertices whose out-degree is greater than or equal to *θ*. Combining Eqs (5) and (7), we obtain:

$$\begin{array}{c} C_{d}=\left(b-1\right)n\cdot P_{out}\left(\theta \right) \end{array}$$

by substituting the number *n*_*d*_ of vertices in dense vertex sets for *n*. By summing up the two costs *C*_*a*_ and *C*_*d*_, we get the claimed communication cost.

Although the exact communication cost of our method in Lemma 3 includes data-dependent terms (*d*_*q*_(*θ*) and *P*_*out*_(*θ*)) and thus is not directly comparable to those of other methods based on single type of messages, we experimentally show that the method achieves smaller amount of communication cost than those of other methods if we choose proper out-degree threshold *θ*_*opt*_.

To choose the optimal out-degree threshold *θ*_*opt*_ which minimizes *C*_*flex*_, FlexGraph computes the costs for all possible threshold values using Lemma 3. The set of possible threshold values consists of the out-degrees of vertices in the input graph, which are less than or equal to the maximum out-degree *d*_*max*_. Given the out-degree distribution of the input graph and an out-degree threshold *θ*, the required data-dependent terms to compute *C*_*flex*_ are computed in constant time. Even though we need additional time to compute the out-degree distribution, the distribution can be approximated using power-law degree distribution in general. Thus, finding the optimal out-degree threshold has *O*(*d*_*max*_) time complexity. Note that *d*_*max*_ is less than few thousands in most real-world graphs.

### Flexible storage format

Another major performance bottleneck is the I/O cost from reading the input graph repeatedly. In general, distributed graph processing systems partition a whole graph into subgraphs, and store the partitioned subgraphs into main or external-memory space of workers. Then, each worker reads the stored subgraphs from main or external-memory space at every iteration. Therefore, reducing the I/O cost to load the edge sets from external-memory space is also valuable to speed up the graph computation. Furthermore, some graph processing systems including FlexGraph, Giraph, and Pregelix cache the subgraphs into main memory space of workers if there is space in the main memory. If the size of the serialized subgraphs decreases, more subgraphs can be cached in the main memory.

**Fig 4:**
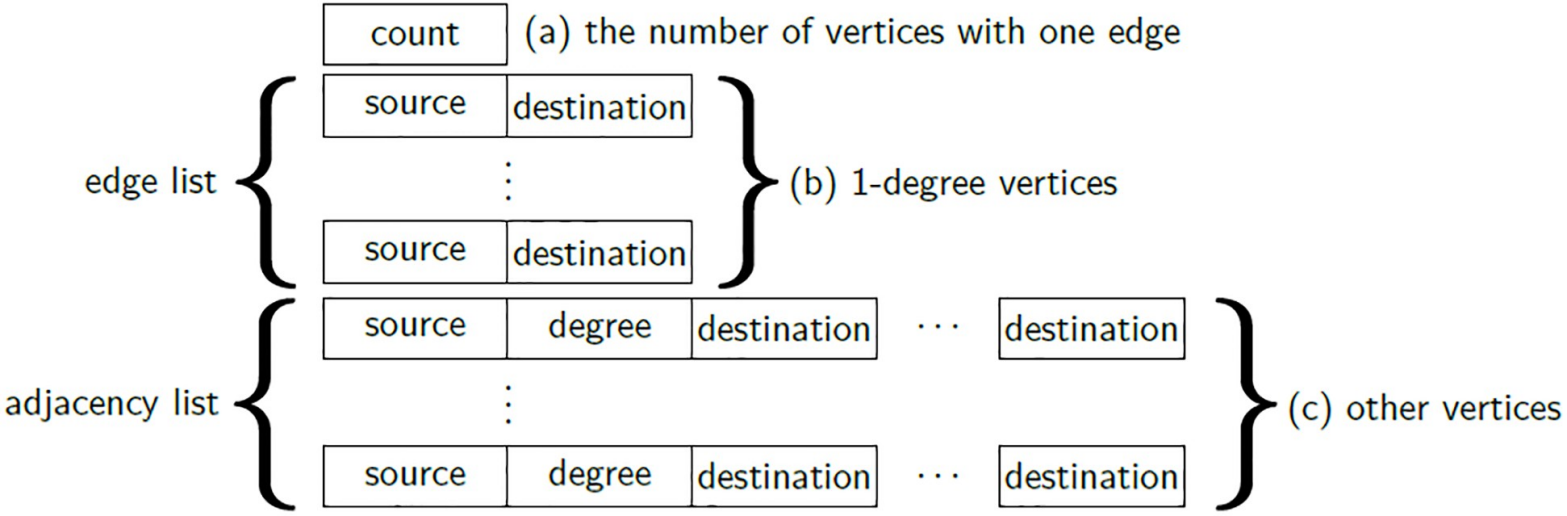
The storage of subgraph in FlexGraph. The format consists of three parts: (a) the number of vertices with out-degree 1, (b) edges of vertices with out-degree 1 in edge list format, and (c) edges of vertices with out-degree ≥ 2 in adjacency list format.

We propose a flexible storage format based on the following two observations. The first observation is that the two widely used storage formats for graph processing, edge list and adjacency list, have their own preferred scenarios. Edge list format stores an edge in a tuple consisting of the source vertex and the destination vertex as illustrated in Fig 4b. On the other hand, adjacency list format groups edges by their source vertex, and stores the grouped edges with out-degree, as shown in Fig 4c. Note that our flexible storage format is designed for disk-based and distributed graph processing systems; thus the id and the out-degree of each source vertex should be stored for loading the partitioned subgraphs from external-memory space of distributed workers. The edge list format is better than the adjacency list format when a vertex with only one edge is stored because degree information is not stored. For storing a vertex with many edges, the adjacency list format is better than the edge list format because the duplicate source vertex ids are eliminated. Lemma 4 verifies that the storage formats have different preferred scenarios.

**Lemma 4**. *If a vertex has d* > 2 *outgoing edges, the size S*_*e*_ *for storing the vertex and its outgoing neighbors in edge list format is larger than the size S*_*a*_ *for that in adjacency list format*.

*Proof*. In the edge list format, the size *S*_*e*_ of a vertex is the sum of (1) the size of source vertex id, and (2) the size of destination vertex id; thus *S*_*e*_ = 2*d* ⋅ *S*_*v*_ where *S*_*v*_ is the size of vertex id in bytes, and *d* is the number of edges. The size *S*_*a*_ of a vertex in adjacency list format is the sum of (1) the size of source vertex id, (2) the size of the number of edges (degree), and (3) the size of destination id list. Therefore, we get *S*_*a*_ = (*d* + 1)*S*_*v*_ + *S*_*l*_ where *S*_*l*_ is the size of the number of edges in bytes. Comparing *S*_*e*_ and *S*_*a*_, we obtain:

$$S_{e}>S_{a}\Leftrightarrow 2d\cdot S_{v}>\left(d+1\right)S_{v}+S_{l}\Leftrightarrow d>1+\frac{S_{l}}{S_{v}}$$

In general, *S*_*l*_ = *S*_*v*_ because both vertex id and degree are represented by an integer; thus the claim holds for all *d* > 2.

**Fig 5:**
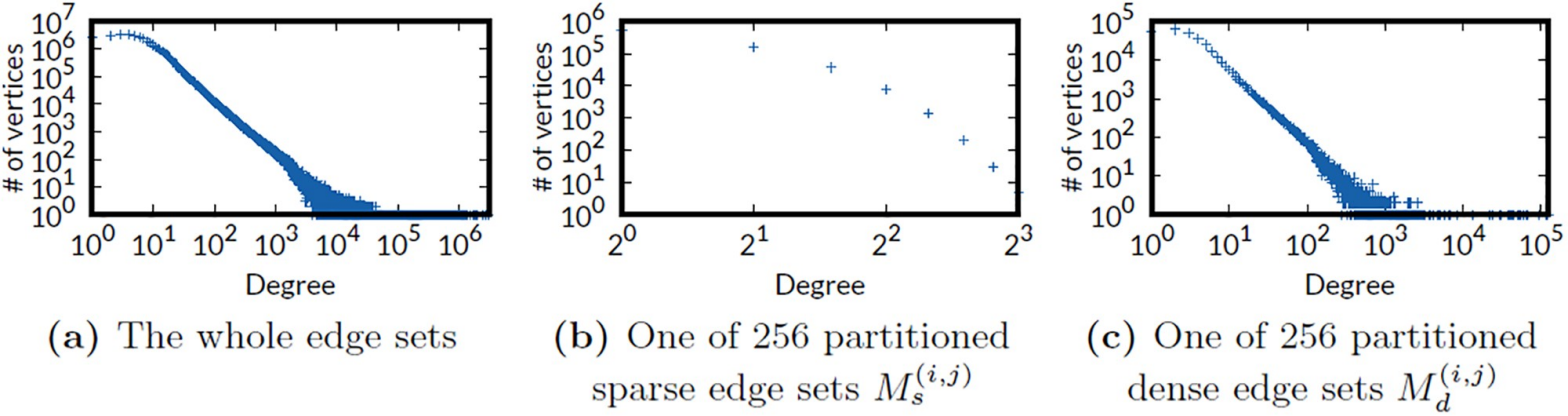
The degree distribution of whole edge set of Twitter graph and one of 256 partitioned edge sets. For all edge sets, there are many vertices with various out-degrees, which implies either of the edge list or the adjacency list format does not efficiently store them.

How many vertices have only one outgoing edge? Our second observation is that the subgraphs divided by FlexGraph still follow skewed degree distributions, regardless of whether the edge sets are sparse or dense. In other words, many degree-one vertices and some high degree vertices coexist in each subgraph. Fig 5 shows the degree distribution of Twitter graph and one sample *M*^(*i*,*j*)^ of 256 partitioned edge sets. In sparse edge set $M_{s}^{\left(i,j\right)}$, more than 549K vertices have only one outgoing edge, and 199K vertices have multiple edges. In dense edge set $M_{d}^{\left(i,j\right)}$, similarly, more than 55K vertices have only one outgoing edge, and 278K vertices have multiple edges. Therefore, using single data format to store edge sets is inefficient.

Our flexible storage format stores a vertex and its outgoing edges as an edge list format if the vertex has out-degree 1; otherwise, they are stored as an adjacency list format to minimize the size of stored subgraphs. Fig 4 illustrates the storage scheme of flexible storage format. The format has three parts: (a) the number of vertices with out-degree 1, (b) edges of vertices with out-degree 1 in edge list format, and (c) edges of vertices with out-degree ≥ 2 in adjacency list format.

### Implementation

In this section, we discuss implementation details of FlexGraph to perform GIM-V, a representative graph mining primitive. As we discussed before, a message passing based graph processing can be implemented in an iterative generalized matrix-vector multiplication *M* ⊗ *v* given a matrix *M* that represents an edge set and a vector *v* that represents a vertex set. Therefore, the graph partitioning process of flexible edge placement can be considered as matrix partitioning in matrix-vector multiplication. In terms of matrix partitioning, we define column assignment and row assignment as grouping columns and rows, respectively, and assign each group to a worker. Note that column assignment is identical to source edge-cut, and row assignment is identical to destination edge-cut. Column assignment incurs no duplication messages because the part of vector *v* required to process the columns in each worker is already in the worker. On the other hand, row assignment incurs no aggregation messages because intermediate result in each worker can be merged into the result vector immediately. Thus, the flexible edge placement in terms of matrix-vector multiplication is the same as a combination of column and row assignment.

**Algorithm 1** Matrix Pre-partitioning in FlexGraph

**Input**: a sparse matrix *M* = {(*i*, *j*, *m*_*i*,*j*_) | 0 ≤ *i*, *j* < *n*}, a vector *v* = {*v*_*j*_ | *j* ∈ {0, ⋯, *n* − 1}}, the number *b* of blocks, a vertex partitioning function *ψ*, a degree threshold *θ*

**Output**: a set $\left\{(M_{s}^{\left(:,q\right)},v_{s}^{\left(q\right)})\;|\;q\in \{0,\cdots ,b-1\}\right\}$ of sparse matrices, a set $\left\{(M_{d}^{\left(p,:\right)},v_{d}^{\left(p\right)})\;|\;p\in \{0,\cdots ,b-1\}\right\}$ of dense matrices

1: **for each** vertex *j* ∈ *v* **do in parallel**

2:  *q* ← *ψ*(*j*)

3:  *d*_*j*_ ← |{*m*_:,*j*_ | *j* ∈ {0, ⋯, *n* − 1}}| // out-degree of *j*

4:  add *v*_*j*_ to *v*^(*q*)^

5:  add *d*_*j*_ to *d*^(*q*)^

6: **for each** matrix element *m*_*i*,*j*_ **do in parallel**

7:  *p* ← *ψ*(*i*)

8:  *q* ← *ψ*(*j*)

9:  add *m*_*i*,*j*_ to *M*^(*p*,*q*)^

10: **for each** triple (*v*^(*q*)^, *M*^(:,*q*)^, *d*^(*q*)^) **do in parallel**

11:  **for each** vertex *j* ∈ *v*^(*q*)^ **do**

12:   **if** *d*_*j*_ < *θ* **then**

13:    add *v*_*j*_ to $v_{s}^{\left(q\right)}$

14:   **else**

15:    add *v*_*j*_ to $v_{d}^{\left(q\right)}$

16:  **for each** matrix block *M*^(*p*,*q*)^ **do**

17:   **for each** matrix element *m*_*i*,*j*_ ∈ *M*^(*p*,*q*)^ **do**

18:    **if** *d*_*j*_ < *θ* **then**

19:     add *m*_*i*,*j*_ to $M_{s}^{\left(p,q\right)}$

20:    **else**

21:     add *m*_*i*,*j*_ to $M_{d}^{\left(p,q\right)}$

22: $M_{s}\leftarrow \{M_{s}^{\left(:,q\right)}\;|\;q\in \{1,\cdots ,b\}\}$

23: $M_{d}\leftarrow \{M_{d}^{\left(p,:\right)}\;|\;p\in \{1,\cdots ,b\}\}$

24: **return** *M*_*s*_, *M*_*d*_

Algorithm 1 describes the matrix pre-partitioning of FlexGraph. Given an input vector *v* and its corresponding matrix *M*, FlexGraph first computes the out-degree of each vertex, and partitions the input vector *v* into *b* sub-vectors (lines 1-5). Then, the input matrix *M* is divided into corresponding *b*^2^ sub-matrices (lines 6-9). After that, FlexGraph divides each sub-vector *v*^(*q*)^ into a sparse sub-vector $v_{s}^{\left(q\right)}$ and a dense sub-vector $v_{d}^{\left(q\right)}$; $v_{s}^{\left(q\right)}$ consists of vertices whose out-degrees are smaller than a threshold *θ*, and $v_{d}^{\left(q\right)}$ consists of vertices whose out-degrees are greater than or equal to *θ* (lines 11-15). Likewise, each sub-matrix *M*^(*p*, *q*)^ is also divided into a sparse sub-matrix $M_{s}^{\left(p,q\right)}$ where each source vertex is in $v_{s}^{\left(q\right)}$, and a dense sub-matrix $M_{d}^{\left(p,q\right)}$ where each source vertex is in $v_{d}^{\left(q\right)}$ (lines 16-21). Then, the sparse sub-matrices $M_{s}^{\left(:,q\right)}$ are grouped by column with index *q*, while the dense sub-matrices $M_{d}^{\left(p,:\right)}$ are grouped by row with index *p*, respectively (lines 22-23).

**Algorithm 2** Iterative Multiplication in FlexGraph

**Input**: a set $\left\{(M_{s}^{\left(:,q\right)},v_{s}^{\left(q\right)})\;|\;q\in \{0,\cdots ,b-1\}\right\}$ of sparse regions, a set $\left\{(M_{d}^{\left(q,:\right)},v_{d}^{\left(q\right)})\;|\;q\in \{0,\cdots ,b-1\}\right\}$ of dense regions

**Output**: a result vector *v*′ = {*v*′^(*q*)^ | *q* ∈ {0, ⋯, *b* − 1}}

1: **repeat**

2:  **for each** ($M_{s}^{\left(:,q\right)},v_{s}^{\left(q\right)},M_{d}^{\left(q,:\right)},v_{d}^{\left(q\right)}$) **do in parallel**

3:   **for each** sub-matrix $M_{s}^{\left(p,q\right)}\in M_{s}^{\left(:,q\right)}$ **do**

4:    $v_{s}^{\left(p,q\right)}\leftarrow {\text{combineAll}}_{b}({\text{combine}2}_{b}(M_{s}^{\left(p,q\right)},v_{s}^{\left(q\right)}))$

5:    **if** p ≠ q **then**

6:     *w*_*p*_ ← the worker that has $v_{s}^{\left(p\right)}$ and $v_{d}^{\left(p\right)}$

7:     send $v_{s}^{\left(p,q\right)}$ to the worker *w*_*p*_

8:     send request for $v_{d}^{\left(p\right)}$ to the worker *w*_*p*_

9:   $r^{\left(q\right)}\leftarrow v_{s}^{\left(q,q\right)}$

10:   **for each** received vector $v_{s}^{\left(q,p\right)}$ **do**

11:    send $v_{d}^{\left(q\right)}$ to the worker that sent $v_{s}^{\left(q,p\right)}$

12:    $r^{\left(q\right)}\leftarrow {\text{combineAll}}_{b}(r^{\left(q\right)}\cup v_{s}^{\left(q,p\right)})$

13:   **for each** sub-region $M_{d}^{\left(q,p\right)}\in M_{d}^{\left(q,:\right)}$ **do**

14:    load the received dense vector $v_{d}^{\left(p\right)}$

15:    $v_{d}^{\left(q,p\right)}\leftarrow {\text{combine}2}_{b}(M_{d}^{\left(q,p\right)},v_{d}^{\left(p\right)})$

16:    $r^{\left(q\right)}\leftarrow {\text{combineAll}}_{b}(r^{\left(q\right)}\cup v_{d}^{\left(q,p\right)})$

17:   $v^{\prime (q)}\leftarrow {\text{assign}}_{b}(v_{s}^{\left(q\right)}\cup v_{d}^{\left(q\right)},r^{\left(q\right)})$

18: **until** convergence

After the pre-partitioning step, the group of sparse sub-matrices $M_{s}^{\left(:,q\right)}$ and the group of dense sub-matrices $M_{d}^{\left(q,:\right)}$ are assigned to a worker. The sparse sub-vector $v_{s}^{\left(q\right)}$ and the dense sub-vector $v_{d}^{\left(q\right)}$ are also assigned to the worker. Algorithm 2 describes the iterative multiplication on the partitioned sub-matrices and sub-vectors. Each worker first multiplies all assigned sparse matrix-vector pairs $\left(M_{s}^{\left(:,q\right)},v_{s}^{\left(q\right)}\right)$, and sends the results to other workers (lines 4 and 7). Then, each worker sends a request for the dense vector $v_{d}^{\left(p\right)}$ to other worker (line 8). Note that the requests for vector values are aggregated into single dense vector request, and the request is sent once to all workers, while PowerGraph and PowerLyra sends massive number of requests. The intermediate result $v_{s}^{\left(p,q\right)}$ is stored in memory space or external-memory space of the worker that has $v_{s}^{\left(p\right)}$. Since each worker has the sparse sub-vector $v_{s}^{\left(q\right)}$ for all corresponding sparse sub-matrices, there is no communication cost to duplicate the sub-vector. After that, each worker sends the requested dense vector $v_{d}^{\left(q\right)}$, and reduces the intermediate result $v_{s}^{\left(q,p\right)}$ into *r*^(*q*)^ (lines 10-12). Then, the dense matrix-vector pairs $M_{d}^{\left(q,:\right)},v_{d}^{\left(p\right)}$ are multiplied (lines 13-16). The multiplication result of each dense matrix-vector pair is combined into *r*^(*q*)^ immediately. Finally, the result vector *v*′^(*q*)^ is computed by applying assign operation on reduced vector *r*^(*q*)^ (line 17). FlexGraph repeats this task until convergence. Note that combineAll_*b*_ and combine2_*b*_ are block operations for combineAll and combine2, respectively; combine2_*b*_(*M*^(*i*,*j*)^, *v*^(*j*)^) applies combine2(*m*_*p*,*q*_, *v*_*q*_) for all *m*_*p*,*q*_ ∈ *M*^(*i*,*j*)^ and *v*_*q*_ ∈ *v*^(*j*)^, and combineAll_*b*_(*X*^(*i*,*j*)^) reduces each row values in *X*^(*i*,*j*)^ into a single value by applying the combineAll operation.

To process a graph whose size exceeds the main memory space of distributed workers, we consider the number of workers, the size of vector, and the main memory space of workers to determine the number *b* of blocks. *b* is set to the number *W* of workers to maximize the parallelism if |v|/ℳ<W, otherwise *b* is set to O(|v|/ℳ) to fit a sub-vector into the main memory of size ℳ. Note that this proper setting for *b* makes FlexGraph scale to the graph whose size exceeds the main memory size. Based on this setting, each worker requires *O*(|*v*|/*b*) of the main memory size; a sub-multiplication should retain sub-vectors $v_{s}^{\left(q\right)}$ and $v_{d}^{\left(q\right)}$ which are subsets of a vector *v*^(*q*)^ whose expected size is *O*(|*v*|/*b*). The sub-matrix $M_{s}^{\left(p,q\right)}$ or $M_{d}^{\left(p,q\right)}$ is stored in the main memory or external memory of a worker. The output size of each sub-multiplication is the size of vector *v*^(*p*)^ which is *O*(|*v*|/*b*).

## Experiments

We perform experiments to answer the following questions:

Q1. How much does FlexGraph improve the performance and scalability compared to the existing systems?
Q2. Does FlexGraph work well on real-world graphs?
Q3. How much does the threshold *θ* affect the performance and the amount of I/O in FlexGraph?
Q4. How much does the flexible storage format affect the performance and the amount of I/O in FlexGraph?
Q5. How much does the vertex partitioning function affect the performance?

### Experimental setting

We implemented FlexGraph on Hadoop, the de-facto standard distributed data processing framework. We use the optimal out-degree threshold *θ*_*opt*_ for each graph, as reported in Table 2. We compare FlexGraph to existing graph processing systems: HybridGraph, Pregelix, GraphX, PowerLyra, Giraph, and Graph/H. HybridGraph is a disk-based graph processing system which properly switches two communication schemes: push and pull. We find the best parameters of HybridGraph from an evaluation script provided by the authors. Pregelix is a disk-based graph processing system which exploits database techniques to achieve the high scalability. We use all of their optimization techniques such as B-tree indexing and optimal join planning. GraphX is a graph processing system based on Spark, a general data processing system. We set the number of partitions to achieve the highest scalability for each dataset, and use 2D grid partitioning method. Giraph is a representative in-memory graph processing system which uses source edge-cut. We use the configuration in [59] such as ByteArrayEdges storage format to achieve highest scalability. PowerLyra is the state of the art in-memory graph processing system implemented in C++. We use 100 as the in-degree threshold as recommended by the authors. Graph/H is an implementation of PowerLyra partitioning scheme (hybrid-cut) in GraphX. We use the same configuration as in PowerLyra. Note that PowerGraph, a well-known graph processing system using vertex-cut, is excluded from the experiment because PowerLyra is based on and outperforms PowerGraph. PEGASUS, a MapReduce-based graph processing system using GIM-V primitive, is also excluded from the experiment because PEGASUS shows incomparable performance. As we discussed before, PEGASUS shuffles the whole input graph for every iteration causing extremely massive communication cost.

**Table 2:** The summary of graphs.

Graph	Vertices	Edges	*θ*_*opt*_	Source
ClueWeb12 (CW12)	6,231,126,594	71,746,553,402	74	Lemur Project
ClueWeb09 (CW09)	1,684,876,525	7,939,647,897	77	Lemur Project
YahooWeb (YW)	720,242,173	6,636,600,779	71	Yahoo!
Twitter (TW)	41,652,230	1,468,365,182	65	Kwak et al. [57]
RMAT-*k* (RM-*k*)	2^*k*^	2^*k*+5^	69-72	Jeon et al. [58]

We use real and synthetic graphs summarized in Table 2. *Twitter* is a who-follows-whom social network crawled in 2010. *YahooWeb*, *ClueWeb09* and *ClueWeb12* are page-level hyperlink networks on the WWW. RMAT [60] is a widely used graph generation model that matches the characteristic of real-world networks. We generate RMAT graphs with parameters *a* = 0.57, *b* = 0.19, *c* = 0.19, and *d* = 0.05 using TegViz [58], a distributed graph generator.

We run our experiments on a cluster of 17 machines; one is a master and the others are for workers. Each machine is equipped with an Intel E3-1240v5 CPU (quad-core, 3.5GHz), 32GB of RAM, and 4 hard disk drives. A machine that is not the master runs 4 workers, and each worker is equipped with 1 CPU core and 6GB of RAM. All the machines are connected via 1 Gigabit Ethernet. Hadoop 2.7.3, Spark 2.2.0 and MPICH 3.0.4 are installed on the cluster.

### Scalability of FlexGraph

We evaluate the scalability of FlexGraph for processing large-scale graphs under two scenarios. We first vary the size of RMAT graphs while fixing the number of machines. Next, we vary the number of machines while fixing the size of graphs. We note that although we use PageRank algorithm for the experiments, we obtain similar conclusions for connected components and single-source shortest path as well.

#### Varying the size of graph

**Fig 6:**
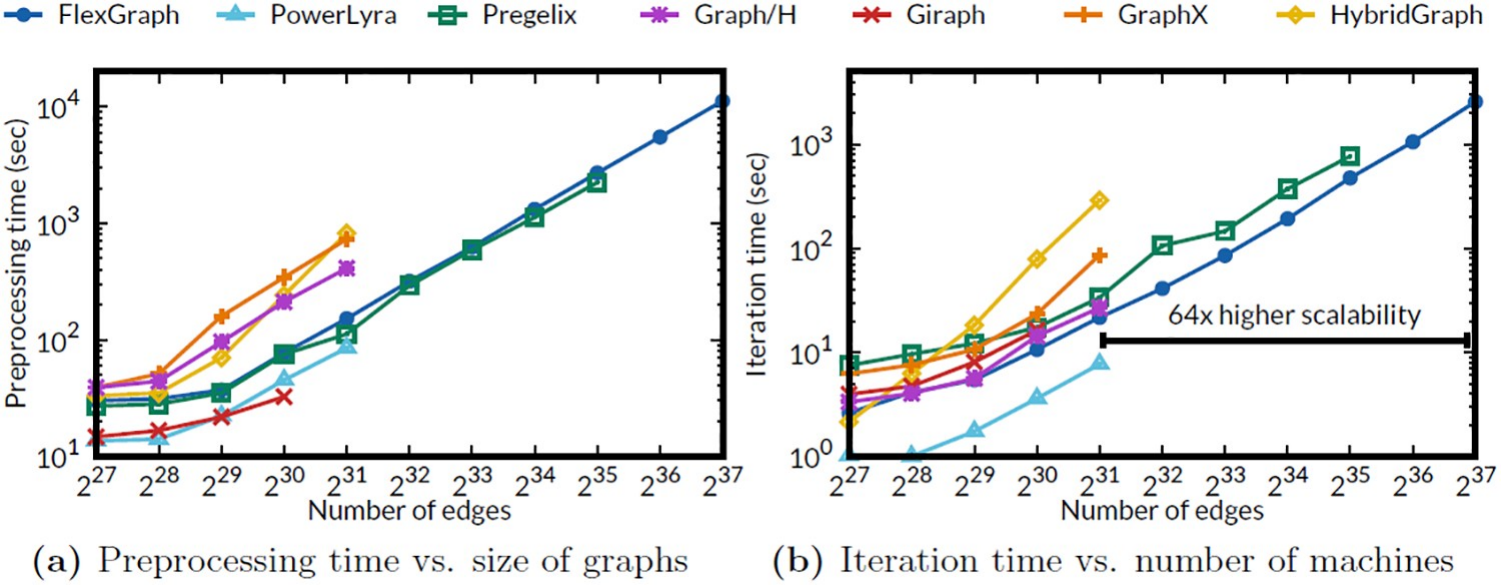
Data scalability of various systems on RMAT graphs. Our proposed FlexGraph is the only system that succeeds on graphs with more than 2^35^ ≈ 34 billion edges.

Fig 6 shows the running time of FlexGraph and competitors (HybridGraph, Pregelix, GraphX, PowerLyra, Giraph, and Graph/H) on RMAT graphs with varying numbers of edges. We report preprocessing time in Fig 6a, and average running time per iteration in Fig 6b. The x-axis is the number of edges and the y-axis is the preprocessing time and the running time in seconds. We emphasize that only FlexGraph succeeds in processing RMAT-32 graph with more than 67 billion edges. The memory-based systems fail on graphs (RMAT-28) with more than 4.1 billion edges due to out of memory error. Among the disk-based systems, FlexGraph outperforms HybridGraph, Pregelix by 3.7 and 1.54 times on average, respectively. Additionally, the disk-based competitors fail on graphs with more than 34 billion edges.

The detailed analysis of the results are as follows. Giraph assumes that a vertex, all of its outgoing edges, and all messages sent to and received from other workers fit into the main memory of a worker. However, this assumption can be easily broken by the power-law degree distribution of real-world graphs; a vertex can have tremendous outgoing edges exceeding the size of main memory, and causes massive number of messages. GraphX uses a vertex-cut partitioning method and copies vertices to multiple machines containing edges incident to the vertices. The edges and the copied vertices are stored in the main memory of each worker, and incur the out of memory error. Even though PowerLyra reduces the number of copied vertices with its hybrid-cut, the size of intermediate data still exceeds the main memory size of each worker. Thanks to the hybrid-cut from PowerLyra, Graph/H shows better performance than GraphX does. However, Graph/H has the same limitation of GraphX, its underlying graph processing framework. We emphasize that there is a large performance gap between Graph/H and PowerLyra, even though they use the same hybrid-cut partitioning scheme. We presume that the main reason of PowerLyra’s performance gain is from its implementation language C++ which does not require time for serialization and deserialization, unlike Java used in Graph/H. HybridGraph requires duplication of all edges to switch communication mechanism, and is tied strictly with range vertex partitioning to exploit graph locality. But copying all edges twice requires massive processing time as input graph size grows. Furthermore, the range partitioning causes extremely unbalanced edge distribution among machines on graphs with skewed degree distributions. Pregelix constructs an additional B-tree based data index for joining vertices and messages from other vertices. The index resides in the main memory of a worker, however, the index size can exceed the size of main memory if a vertex has numerous incoming edges.

**Fig 7:**
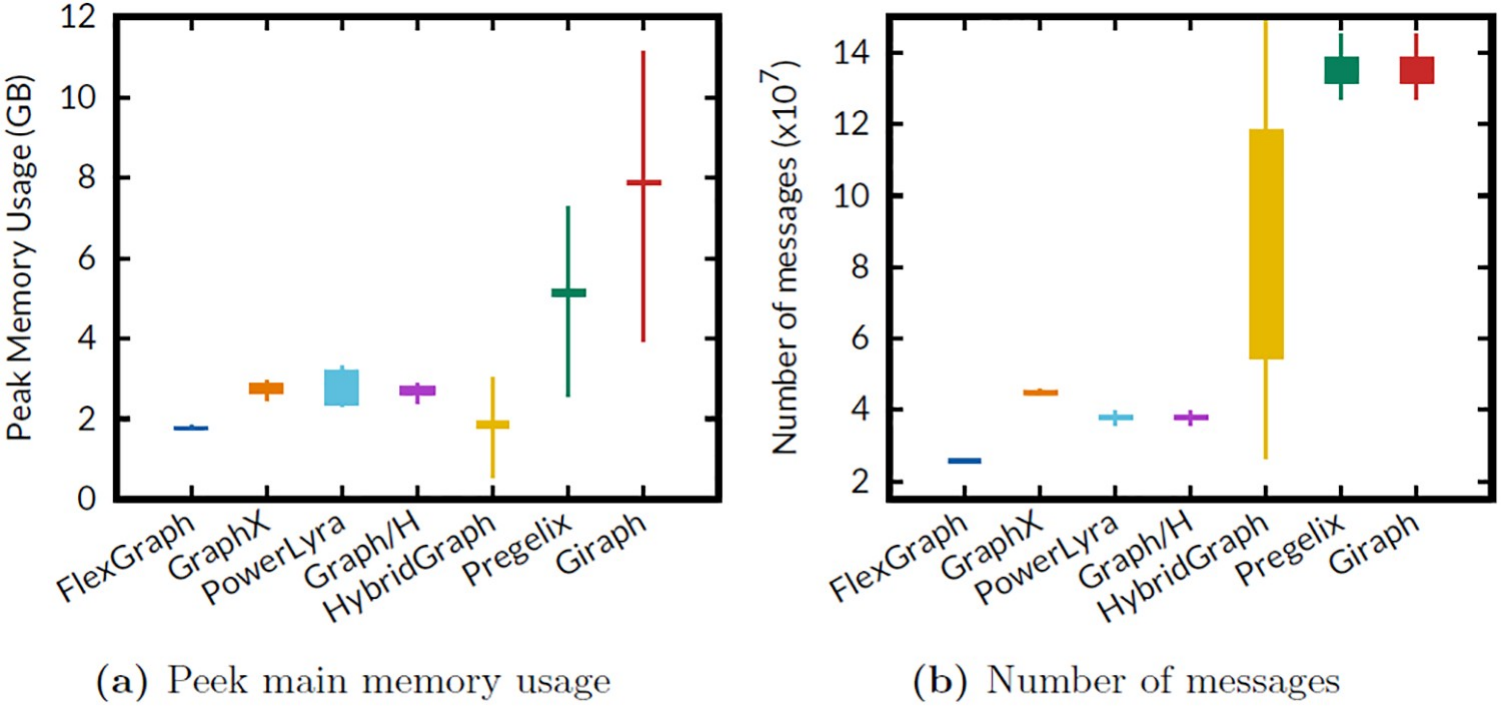
Workload distribution of various systems. (a) FlexGraph achieves the lowest memory usage and almost uniform memory usage distribution. (b) Thanks to the flexible edge placement, FlexGraph shows the smallest number of message exchanges between workers.

We measure the peak main memory usage of a worker and the number of messages between workers to verify the underlying reasons of scalability and performance difference, when running PageRank algorithm on RMAT-24 graph. Fig 7a and 7b are box-and-whisker plots showing the peak memory usage for each worker, and the number of messages between workers, respectively. Thanks to the flexible edge placement and the flexible storage format, FlexGraph achieves the lowest memory usage, the smallest number of messages, and almost uniform memory usage distribution. The systems based on vertex-cut and hybrid-cut, such as GraphX, PowerLyra, and Graph/H also achieve almost uniform memory usage distribution. However, these systems use more memory than FlexGraph does because FlexGraph compresses partitioned subgraphs using the flexible storage format, even though each worker holds the subgraphs in main memory. Comparing Graph/H with PowerLyra, we observe that the difference of number of messages is negligible; thus the main performance difference may come from the language difference, Java and C++. The systems using source edge-cut such as HybridGraph, Pregelix, and Giraph show poor distribution of memory usage due to skewed degree distributions of real world graph. Especially, the largest memory usage of a worker in Giraph is 2.8 times larger than its smallest memory usage. Note that HybridGraph shows highly skewed distribution of number of messages because HybridGraph uses range partitioning. On the other hand, HybridGraph does not use much memory since it stores the intermediate data including messages from other workers in disk.

#### Varying the number of machines

**Fig 8:**
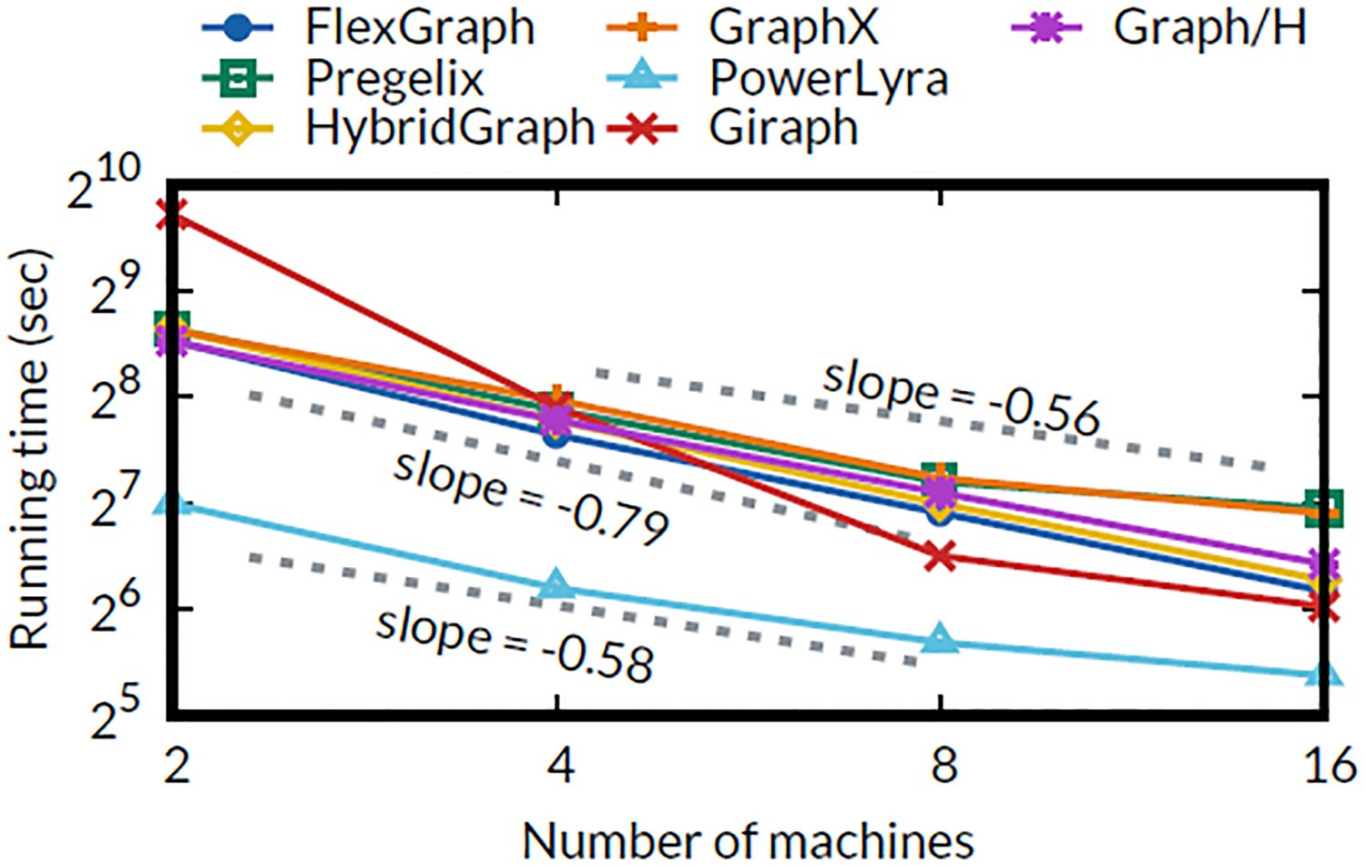
Machine scalability of various systems. FlexGraph and HybridGraph show better machine scalability (slope = -0.79) than Pregelix, GraphX, Graph/H (slope = -0.56), and PowerLyra (slope = -0.58).

We evaluate machine scalability of FlexGraph and competitors by running the PageRank algorithm on RMAT-24 with varying numbers of machines. Fig 8 shows the running time of FlexGraph and competitors varying the number of machines from 2 to 16 in log-log scale. Giraph shows the steepest slope because its running time on 2 machines is extremely slow due to pause time from massive garbage collection. PowerLyra achieves the highest performance compared to other methods, but shows limited scalability (slope = -0.58). Pregelix, GraphX, and Graph/H show poor scalability (slope = -0.56) on 16 machines because of waiting time for network operations in join stage. FlexGraph and HybridGraph scale up better (slope = -0.79) than other methods by avoiding the blocking time for I/Os via overlapping computation with I/O operations, and optimized data transfer. Even though both FlexGraph and HybridGraph achieve similar machine scalability, we emphasize that FlexGraph consistently outperforms HybridGraph in all experiments.

### Experiments on real-world graphs

We test three graph algorithms, PageRank, single-source shortest path (SSSP), and connected components (CC), to evaluate the overall performance of FlexGraph on four real-world graphs in Table 2. For PageRank, the number of iterations is set to 10; for SSSP and CC, iterations end on convergence. The vertex with the highest out-degree in each graph is set to the source vertex for SSSP.

**Fig 9:**
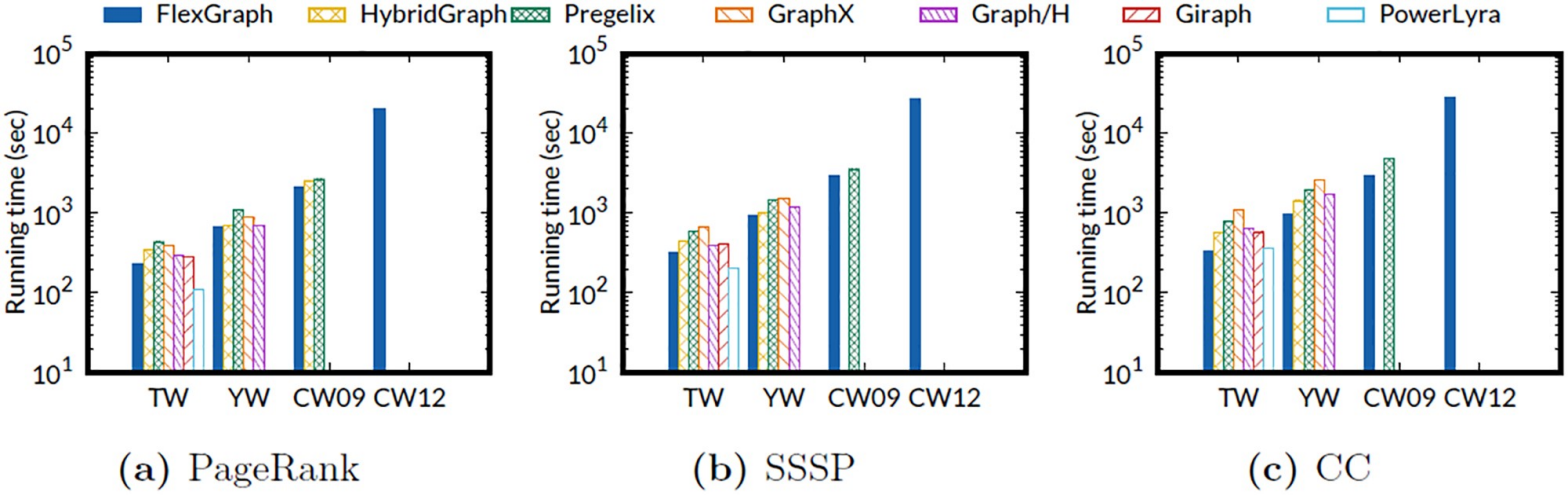
Running times of various algorithms on real-world graphs. FlexGraph is the only method which succeeds in processing ClueWeb12 graph for all graph computations.

Fig 9 shows the results. FlexGraph is the only method that succeeds in processing ClueWeb12. PowerLyra is faster than FlexGraph for the smallest graph TW in PageRank and SSSP computations, and is comparable to FlexGraph in CC computation. As discussed in scalability experiments, our conjecture is that this fast running time comes from PowerLyra’s implementation language C++ which allows faster execution than Java which all other methods use. However, PowerLyra fails in processing web-scale graphs (YW, CW09, and CW12) because the size of intermediate data for edges, vertices, and states of masters and mirrors exceeds the size of aggregated memory of all machines, causing out of memory error. As in scalability experiments, there is a large performance gap between PowerLyra and Graph/H which use the same partitioning scheme. Giraph, another memory-based method, also fails in processing the web-scale graphs because of memory pressure from skewed degree distribution which is common in real-world graphs. GraphX processes YahooWeb graph thanks to disk utilization of Spark, the underlying method of GraphX, but fails in processing ClueWeb09 graph suffering from increased memory pressure caused by massive number of RDD partitions. Note that GraphX loads all intermediate data for a subgraph including messages, copied vertex states, and additional data structure for vertex indexing, incurring the large number of RDD partitions, while FlexGraph streams intermediate data as messages. Even if we increase the number of subgraphs to reduce the memory pressure, the size of each RDD partition does not decrease because the number of copied vertices increases. HybridGraph fails in processing the ClueWeb09 graph due to I/O inefficiency from accessing all vertices and edges, even though SSSP and CC need to process only a portion of vertices in each iteration.

### Effect of threshold *θ*

**Fig 10:**
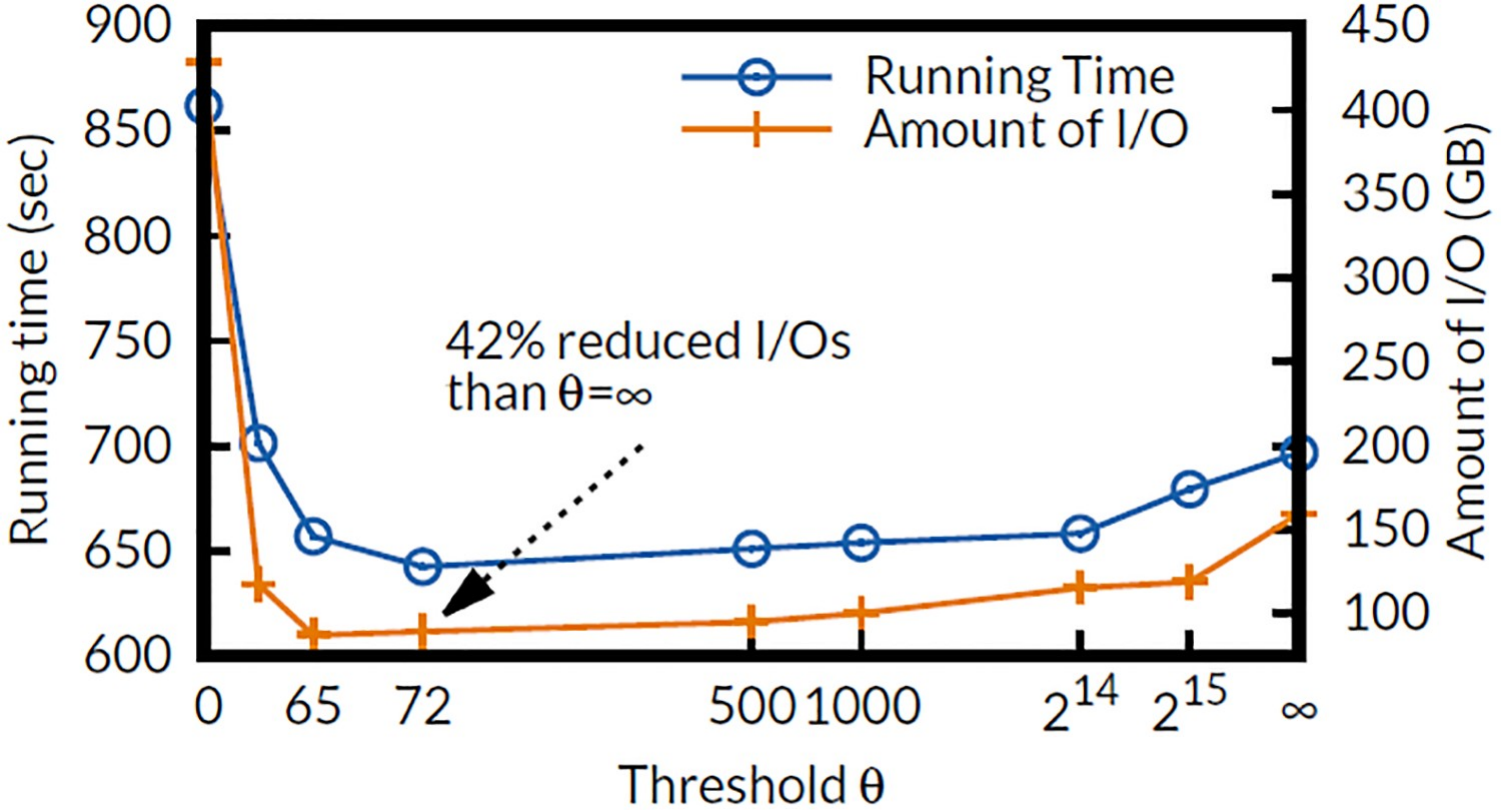
Effect of threshold *θ* on the running time and the amount of I/O. FlexGraph shows the fastest running time when *θ* = 72 with 42% reduced amount of I/O compared to when *θ* = ∞.

To test the effect of the threshold *θ*, we run the PageRank algorithm of FlexGraph for 30 iterations on Twitter graph varying *θ*. Fig 10 presents the running time and the amount of I/O. In general, FlexGraph with *θ* = ∞ shows better performance than FlexGraph with *θ* = 0 does because real-world graphs are extremely sparse. FlexGraph achieves the fastest running time with *θ* = 72, showing 42% decreased amount of I/O compared to *θ* = ∞, from 154GB to 89GB.

### Effects of flexible storage format

**Fig 11:**
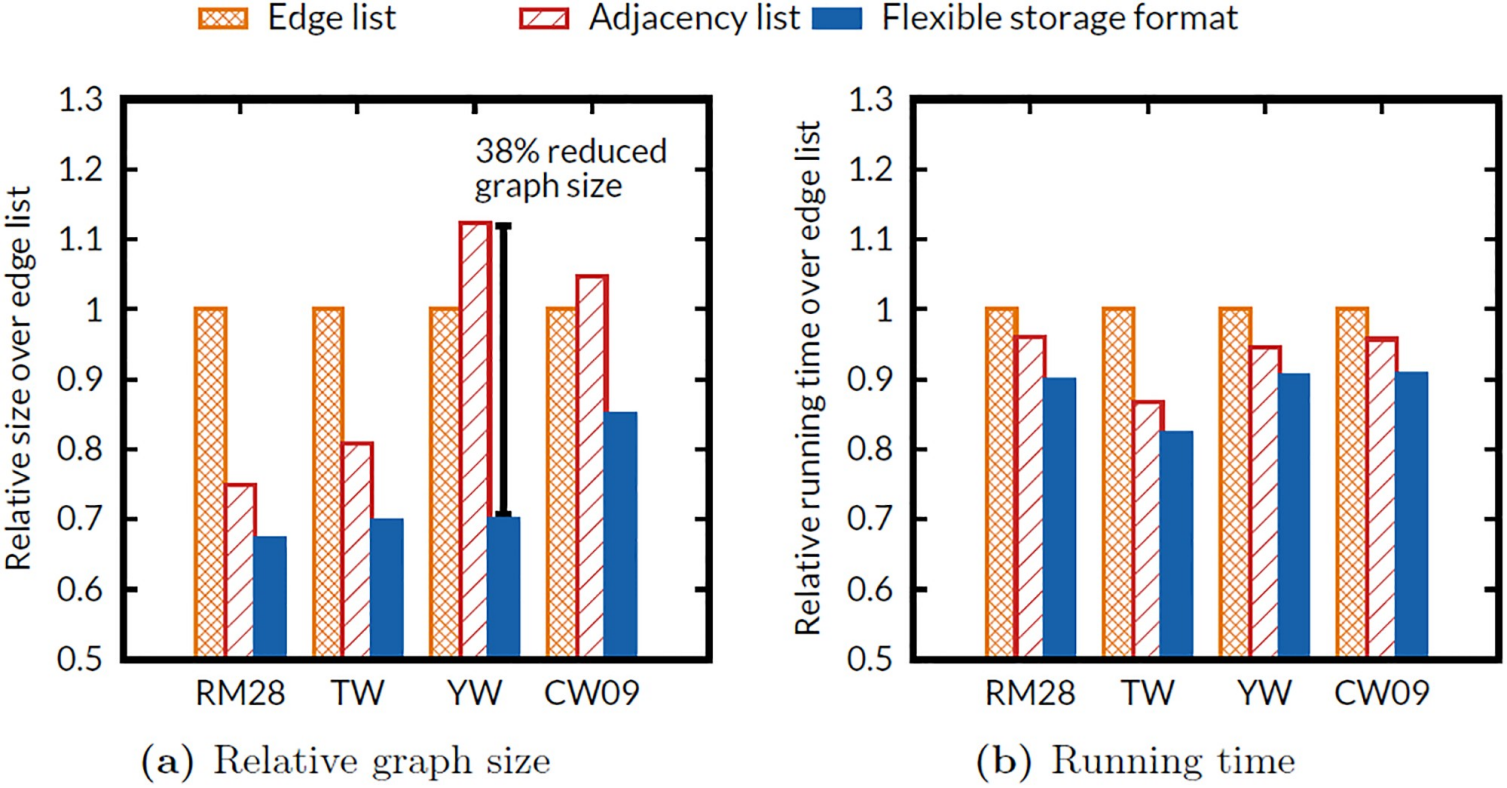
Effects of different edge storage formats on various graphs. (a) Relative size of graphs stored in different storage formats. (b) Running time of FlexGraph using different storage formats. The flexible storage format shows the smallest data size, and contributes to the fastest running time of graph processing by reducing I/O cost.

We evaluate the size of subgraphs and the running time of PageRank algorithm for 10 iterations varying data formats: (1) edge list, (2) adjacency list, and (3) flexible storage format. We use a synthetic graph and three real-world graphs. *YahooWeb* (YW) and *ClueWeb09* (CW09) are sparse real-world graphs whose density |*M*|/|*v*|^2^ is smaller than 10^−7^, while *Twitter* (TW) and *RMAT-28* (RM28) are dense graphs whose density is larger than 10^−7^. As discussed before, the edge list format is appropriate for a sparse graph which has many vertices with out degree 1, while the adjacency list format is appropriate for a dense graph. Fig 11 verifies the relation between the graph density and the efficiency of each graph storage format. For sparse graphs (YW and CW09), the edge list format shows a smaller size than the adjacency list format does. For dense graphs, the adjacency list format shows a smaller size than the edge list format does. Our proposed flexible storage format shows the smallest data size for all graphs, and reduces the size of graphs up to 38% compared to the adjacency list format.

Interestingly, for running time comparison, graph computation with adjacency list is faster than that with edge list for all graphs, although the size of subgraphs in edge list is smaller than that in adjacency list for YahooWeb and ClueWeb09. The underlying reason is that adjacency list format improves cache hit ratio to access source vertex information because subgraphs in adjacency list format are sorted according to their source vertices. In large-scale graph processing, cache hit ratio is one of important factors for performance [61, 62]. Flexible storage format achieves the shortest running time by taking the best of the both formats.

### Effects of vertex partitioning functions

**Fig 12:**
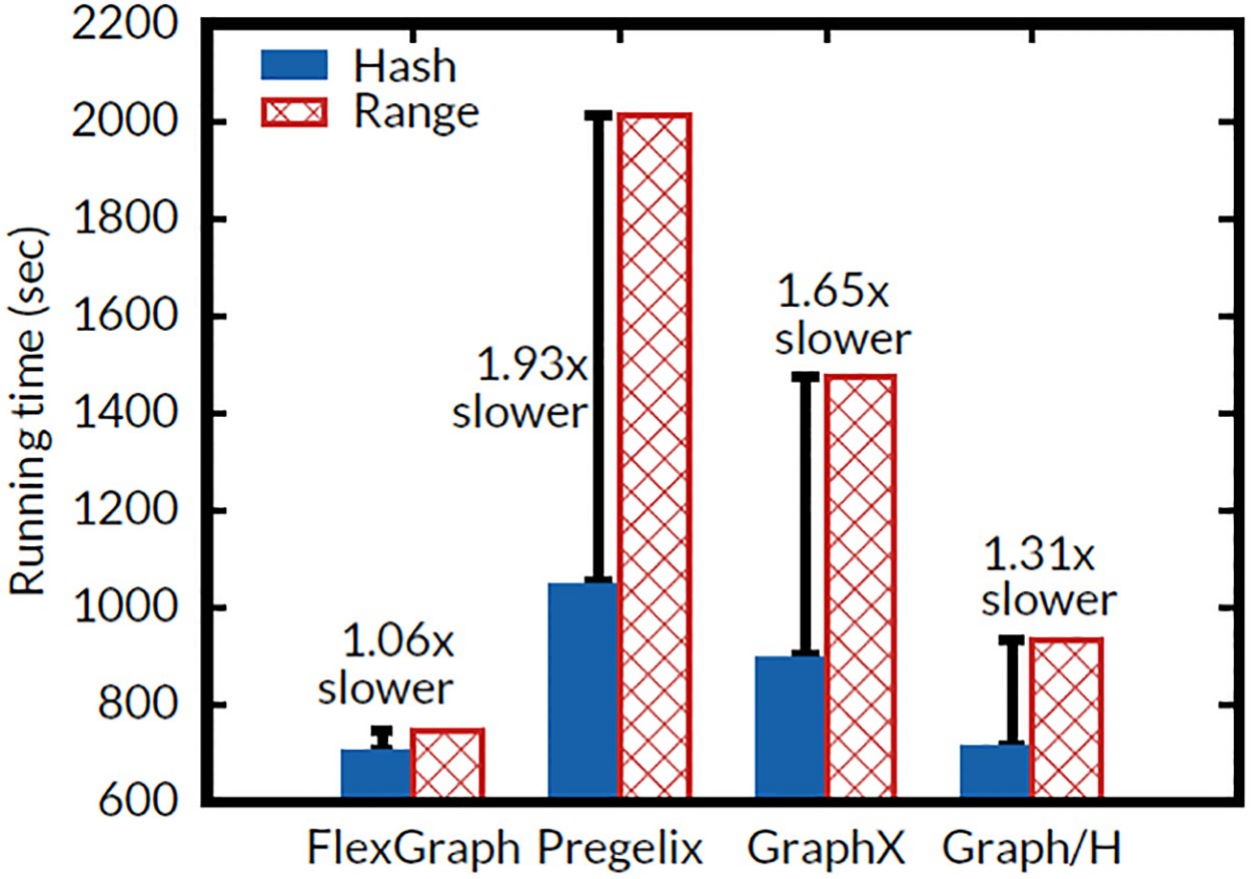
Running times of PageRank on YahooWeb graph with varying vertex partitioning functions. Our proposed FlexGraph is the only method that is robust to vertex partitioning functions.

As reported by Khayyat et al. [63], vertex partitioning functions significantly affect the running time of graph processing. To show the effect of vertex partitioning functions on FlexGraph and competitors, we evaluate the running time of PageRank for 10 iterations on YahooWeb with two popular partitioning functions: (1) hash partitioning and (2) range partitioning. Methods that fail to process YahooWeb are omitted. In the hash partitioning, each vertex is assigned to a worker by a random hash function; thus degree distribution of each partition is similar to that of other partition. In the range partitioning, each vertex partition is a sequential range of vertex ids, and assigned to a worker. Massive high-degree vertices can be assigned to a worker if they have consecutive vertex ids, and cause performance bottleneck due to poor load balancing. HybridGraph is also omitted since its implementation supports only range partitioning to exploit graph locality, though it succeeds in processing YahooWeb. Fig 12 shows the effect of vertex partitioning functions. Note that our proposed FlexGraph is the only method that is robust to vertex partitioning functions thanks to the destination edge-cut for edges from high out-degree vertices which reduces the effect of concentration of high out-degree vertices. Pregelix with range partitioning suffers from unbalanced computation due to skewed degree distribution [64]. On GraphX, similarly, range partitioning results in worse performance than hash partitioning, but for a different reason; GraphX requires a larger number of partitions for range partitioning than for hash partitioning to avoid out of memory error, which is occurred by high degree vertices congregated on a worker. The large number of partitions increases network I/Os. Nonetheless, the performance gap of range partitioning and hash partitioning on GraphX is relatively smaller than that on Pregelix thanks to 2D block partitioning of GraphX. Graph/H, which uses hybrid-cut on GraphX system, shows smaller time difference than Pregelix and GraphX do. However, the time difference of Graph/H is larger than that of FlexGraph because Graph/H with range partitioning also requires a large number of partitions, similarly to GraphX.

## Conclusion

We propose FlexGraph, a scalable graph mining method on distributed systems. FlexGraph reduces communication and I/O costs in distributed graph computation by two ideas: (1) flexible edge placement, and (2) flexible storage format. The flexible edge placement suppresses the number of messages between workers by exploiting different edge placement policies based on types of vertices. The flexible storage format reduces the size of partitioned subgraphs which is important in disk-based graph mining systems. FlexGraph shows up to 64× larger scalability than existing distributed memory based methods, and consistently outperforms existing disk-based ones.
